# Association of Prenatal Ozone Exposure with Fetal Growth and Birth Outcomes: Roles of Maternal Inflammation and Metabolic Dysregulation

**DOI:** 10.3390/toxics13110983

**Published:** 2025-11-15

**Authors:** Zexin Yu, Chunyan Wang, Yueyi Lv, Mengjun Chang, Hao Wang, Yunyun Du, Xianjia Li, Jin Ji, Suzhen Guan

**Affiliations:** Ningxia Key Laboratory of Environmental Factors and Chronic Disease Control, School of Public Health, Ningxia Medical University, Yinchuan 750004, China; zexin_yu111@163.com (Z.Y.); wangcy_0225@163.com (C.W.); 15091470948@163.com (Y.L.); changmengjun777@163.com (M.C.); 18615217356@163.com (H.W.); dyy202323@163.com (Y.D.); 13466884661@163.com (X.L.); j637256869@163.com (J.J.)

**Keywords:** prenatal ozone exposure, air pollution, oxidative stress, adverse pregnancy outcomes, gestational diabetes mellitus (GDM), inflammatory biomarkers, fetal growth

## Abstract

Prenatal ozone (O_3_) exposure may trigger systemic inflammation and oxidative stress. These effects could contribute to adverse pregnancy outcomes. We conducted a prospective cohort study involving 235 pregnant women in Ningxia, China. Maternal O_3_ exposure during pregnancy and prior to conception was assessed using high-resolution spatiotemporal models. Multivariable logistic and linear regression analyses were employed to evaluate the associations between O_3_ exposure and adverse pregnancy outcomes. Mediation and interaction models were further applied to examine the potential modifying roles of gestational diabetes mellitus (GDM) and inflammatory biomarkers. In multivariable analyses adjusted for maternal and environmental covariates, higher prenatal O_3_ exposure was significantly associated with an increased risk of preterm birth (PTB) (OR = 1.24, 95% CI: 1.05~1.45, *p* = 0.010) and low birth weight (LBW) (OR = 1.29, 95% CI: 1.09~1.54, *p* = 0.004). Similarly, elevated maternal SAA and CRP levels were positively associated with these adverse pregnancy outcomes (*p* < 0.05). Notably, higher TNF-α levels were inversely associated with the risks of PTB (OR = 0.15, 95% CI: 0.03~0.85, *p* = 0.032) and LBW (OR = 0.05, 95% CI: 0.01~0.39, *p* = 0.005). IL-17A levels were inversely associated with neonatal length-for-age Z scores (β = −0.28, 95% CI: −0.55~−0.01, *p* = 0.043). Our findings suggest that prenatal O_3_ exposure is associated with increased risks of PTB and LBW. Alterations in systemic inflammatory markers and metabolic dysfunction during pregnancy were related to adverse pregnancy outcomes and fetal growth deficits, but they did not mediate these associations, with O_3_ remaining an independent predictor after adjustment.

## 1. Introduction

In recent years, growing attention has been directed towards the potential adverse effects of air pollution on maternal and fetal health. Extensive epidemiological studies have consistently demonstrated that prenatal exposure to air pollutants can exert significant detrimental impacts on both mothers and their newborns. According to a global burden-of-disease analysis, nearly 6 million preterm births and almost 3 million low-birth-weight infants in 2019 were attributable to air pollution. Among these pollutants, ozone (O_3_) has gained increasing attention for its association [1,2,3,4].

O_3_ is a highly oxidative atmospheric pollutant formed through photochemical reactions between nitrogen oxides (NO_x_) and volatile organic compounds (VOCs) [5]. Currently, ground-level O_3_ concentrations have been steadily increasing in many regions globally, making O_3_ pollution a significant risk factor threatening human health worldwide [6]. O_3_ exerts profound impacts on maternal and fetal health via multiple biological mechanisms, primarily by inducing oxidative stress and triggering inflammatory responses [7]. Several experimental studies have observed that O_3_ exposure can elevate reactive oxygen species (ROS) production and up-regulate oxidative damage markers such as malondialdehyde (MDA) and 8-hydroxy-2′-deoxyguanosine (8-OHdG) in maternal or offspring tissues, and also increase pro-inflammatory cytokines (e.g., IL-6, CRP) [8,9,10,11,12].

Furthermore, chronic inflammation and oxidative stress are considered potential contributors to insulin resistance during pregnancy. These processes may mediate the pathogenesis of gestational diabetes mellitus (GDM) by disrupting insulin signaling pathways [13,14,15]. Several studies have confirmed that prenatal exposure to O_3_ is significantly associated with an increased risk of GDM [3,16]. As a common metabolic disorder during pregnancy, the global incidence of GDM continues to rise and has been clearly linked to both short- and long-term adverse health outcomes for mothers and offspring [17]. Given its central role in maternal glucose metabolism, GDM may not only mediate the effects of prenatal O_3_ exposure on fetal growth and pregnancy outcomes but also modify the susceptibility of certain subgroups, making it an important focus for our analysis.

While several studies have preliminarily revealed the effects of O_3_ exposure on pregnancy outcomes, research on the health risks of O_3_ during pregnancy remains relatively limited compared to pollutants such as PM_2.5_. Few studies have simultaneously examined the modifying effects of GDM and systemic inflammation on the relationship between O_3_ exposure and maternal and neonatal outcomes, highlighting an important gap that our study aims to address. This study, based on a mother–infant cohort from Ningxia, China, aims to evaluate the associations between prenatal O_3_ exposure and adverse pregnancy outcomes, and to further investigate the mediating and modifying roles of maternal metabolic and inflammatory states. By systematically assessing the impact of prenatal O_3_ exposure on pregnancy outcomes and fetal development, this study provides scientific evidence to guide targeted interventions and enhance air pollution control strategies to promote maternal and infant health.

## 2. Materials and Methods

### 2.1. Study Population and Data Collection

This prospective cohort study was conducted from February to September 2023 at the obstetrics outpatient clinic of a tertiary hospital in Ningxia, China. Participants were singleton pregnant women who attended routine prenatal care and delivered at the same hospital. Women were enrolled during early pregnancy (between 8 weeks 0 days and 13 weeks 6 days of gestation) at their first prenatal visit.

Inclusion criteria were: maternal age between 18 and 43 years, absence of major infectious or chronic non-communicable diseases before pregnancy, no residential relocation during pregnancy, and availability of complete follow-up data. Participants with missing residential information (n = 10), a pre-pregnancy diagnosis of psychiatric disorder (n = 1), or absence of GDM diagnostic data (n = 5) were excluded during data cleaning. Therefore, no imputation was performed, and analyses were based on complete cases. A total of 235 pregnant women met the criteria and were included in the final analysis. All participants had complete data on inflammatory biomarkers and perinatal outcomes.

Study data were primarily collected through on-site questionnaires covering maternal lifestyle, health behaviors, sleep quality [18], family satisfaction [19], and basic demographic characteristics. The validated Chinese versions of standardized instruments were used, including the Pittsburgh Sleep Quality Index (PSQI) and the Family APGAR scale. The questionnaires were administered and guided by uniformly trained research staff in the fetal monitoring unit to ensure accuracy and data consistency. Venous blood samples were collected during mid-pregnancy (between 14 weeks 0 days and 27 weeks 6 days of gestation) for inflammatory biomarker assays. Clinical information, including gestational weight gain, pregnancy complications, mode of delivery, and neonatal birth measurements, was extracted from the hospital’s electronic health record system to supplement perinatal outcome data. Written informed consent was obtained from all participants at recruitment.

The study protocol was approved by the Ethics Committee of Ningxia Medical University (approval number: Ning Yi Da Ethics No. 2022·007) and conducted in accordance with the ethical principles of the Declaration of Helsinki.

### 2.2. Air Pollutant Exposure Assessment

O_3_ exposure assessment was conducted using a high-resolution spatiotemporal model that integrated satellite remote sensing data, meteorological parameters, and ground monitoring observations through geographic information system (GIS) techniques. Daily maximum 8 h average O_3_ concentrations (MDA8 O_3_) were estimated at a 500 m × 500 m spatial resolution across the study area. Residential addresses were extracted from the participants’ medical records and geocoded into corresponding latitude and longitude coordinates. Based on the expected delivery date and actual gestational age at birth, the precise pregnancy exposure window was determined to match exposure periods with gestational stages. Using ArcMap 10.7 software, each geocoded residence was assigned a 1 km circular buffer to represent the surrounding exposure environment. Given that the O_3_ prediction model had a spatial resolution of 500 m × 500 m, the 1 km buffer was applied to capture local spatial variability and reduce exposure misclassification. The mean O_3_ concentration within this buffer over the entire pregnancy was calculated as the individual exposure level. This method was also applied to calculate pregnancy exposures to other environmental variables, including ALAN, NDVI, NO_2_, PM_2.5_, rainfall, and temperature, all assessed using similar spatiotemporal models. The performance of the O_3_ model was evaluated using cross-validation, yielding satisfactory accuracy (cross-validated R^2^ ≈ 0.70), indicating reliable exposure estimation. O_3_ exposure was treated as a continuous variable in multivariable regression analyses and was also categorized into quintiles (Q1–Q5), with Q1 representing the lowest exposure level (median = 105.17 µg/m^3^) and Q5 representing the highest exposure level (median = 111.37 µg/m^3^), for comparative analyses.

### 2.3. Outcomes

All study participants underwent a standardized 75 g oral glucose tolerance test (OGTT) during mid-pregnancy, with venous blood samples collected at fasting, 1 h, and 2 h to measure glucose levels. GDM was diagnosed according to the 2010 criteria established by the International Association of Diabetes and Pregnancy Study Groups (IADPSG), with diagnosis confirmed if glucose levels met or exceeded any of the following thresholds: fasting glucose ≥ 5.1 mmol/L, 1 h glucose ≥ 10.0 mmol/L, or 2 h glucose ≥ 8.5 mmol/L [20].

PTB was defined as birth prior to 37 weeks of gestation, and LBW was defined as birth weight less than 2500 g. Fetal growth was evaluated using the length-for-age Z score (LAZ) and weight-for-age Z score (WAZ), standardized indicators of neonatal growth and development calculated based on INTERGROWTH-21st reference values for gestational age and sex [21].

### 2.4. Covariates

Covariates were selected based on prior evidence and their potential confounding effects, and were classified into three categories: demographic and socioeconomic factors, behavioral and lifestyle factors, and environmental factors. Demographic and socioeconomic factors included maternal age, pre-pregnancy BMI and health status, parity, mode and season of conception, education level, per capita monthly income, and residential address. Behavioral and lifestyle factors comprised gestational weight gain, sleep quality, and family function assessed by the APGAR score. Environmental factors encompassed outdoor artificial light at night (ALAN), normalized difference vegetation index (NDVI), NO_2_, PM_2.5_, rainfall, and temperature. All covariates were incorporated into multivariable regression models to adjust for potential confounding in the associations between prenatal exposures and maternal and neonatal outcomes.

### 2.5. Statistical Analysis

Descriptive statistics were first performed for the demographic characteristics of the overall sample and for neonatal PTB and LBW status. Continuous variables were tested for normality; non-normally distributed data were retained in their original form and summarized as medians with interquartile ranges [M (Q1, Q3)]. Group comparisons were conducted using the Kruskal–Wallis test or the Mann–Whitney U test, as appropriate. Categorical variables were expressed as frequencies and percentages [n (%)], and differences across groups were assessed using the χ^2^ test or Fisher’s exact test. All statistical tests were two-sided, and a *p* < 0.05 was considered statistically significant.

To investigate the association between prenatal O_3_ exposure and adverse pregnancy outcomes, PTB and LBW were included as binary outcome variables. All inflammatory biomarkers were log_2_-transformed to improve data distribution. Logistic regression models were constructed to assess the relationship between O_3_ exposure and the risk of PTB and LBW, and to explore potential mechanistic pathways. Univariate logistic regressions were first conducted to identify variables associated with the outcomes. Subsequently, multivariable models were developed to evaluate the independent effects of O_3_ exposure, GDM, and inflammatory biomarkers on the outcomes after adjusting for potential confounders. A stepwise adjustment strategy was employed across three models: Model 1 was unadjusted; Model 2 adjusted for maternal demographic and behavioral characteristics; and Model 3 further accounted for social context and additional environmental exposures. Model performance was evaluated using the Nagelkerke pseudo-R^2^ statistic to assess the overall goodness of fit in R. All statistical analyses were performed using R 4.4.2 software.

Mediation analyses were conducted using the lavaan package in R software. To explore potential mechanisms, this study further conducted mediation analyses, including both parallel and sequential mediation models. GDM was specified as the first-stage mediator (M_1_), and maternal inflammatory biomarkers were treated as second-stage mediators (M_2_), constructing path models in which prenatal O_3_ exposure influenced PTB and LBW through single or multiple mediating pathways. Indirect and direct effects, along with their 95% confidence intervals, were estimated using non-parametric bootstrapping with 5000 resamples.

Given the limited statistical significance of the mediation pathways, interaction analyses were additionally performed. Interaction terms between O_3_ and GDM, as well as between O_3_ and inflammatory biomarkers, were included in the models to assess whether maternal metabolic or immune status modified the association between O_3_ exposure and adverse pregnancy outcomes. To minimize potential bias from overadjustment or pathway masking, only maternal demographic and behavioral covariates were included in the mediation and interaction models. Other environmental pollutants that were highly correlated with O_3_ were excluded to reduce multicollinearity and ensure robust estimation of the interaction effects.

Finally, to evaluate the associations between prenatal O_3_ exposure, GDM, inflammatory biomarkers, and fetal growth, multivariable linear regression analyses were conducted for LAZ and WAZ. Each continuous outcome was analyzed separately using a three-step progressive adjustment strategy, consistent with previous models.

## 3. Results

### 3.1. Group Differences in Adverse Pregnancy Outcomes

A total of 235 pregnant women were included in this study, with ages ranging from 18 to 43 years and an average maternal age at delivery of 31 years. Participants were grouped based on the occurrence of PTB and LBW, and maternal mid-pregnancy characteristics were compared between groups.

Data on birth status (i.e., preterm vs. term births) and study population characteristics were available from 235 pregnant women (Table 1). Eleven percent (n = 27) experienced PTB. The median prenatal O_3_ exposure was slightly higher in the PTB group (109.17 µg/m^3^) than in term births (108.60 µg/m^3^), but this difference did not reach statistical significance (*p* = 0.053); no other variables differed significantly between groups. In the LBW analysis (Table S1 in the Supplement), 24 newborns (10.21%) were identified as LBW. Median O_3_ exposure was significantly higher in the LBW group (109.63 µg/m^3^) compared with non-LBW infants (108.57 µg/m^3^; *p* = 0.005), while no other variables showed significant differences (*p* > 0.05).

### 3.2. Identification of Key Variables Influencing PTB and LBW: Results from Logistic Regression

#### 3.2.1. Identification of Factors Influencing Preterm Birth

**Figure 1:**
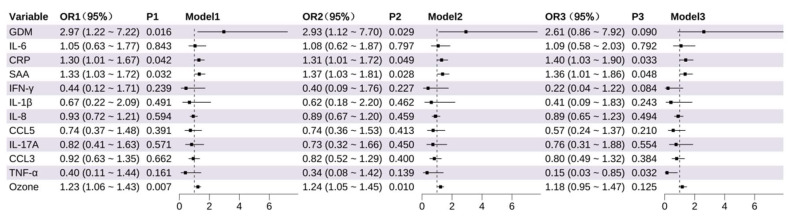
Association of Inflammatory Markers and Environmental Factors With Preterm Birth (PTB). The odds of PTB per increase in log_2_ concentrations (and 95% CIs) of each inflammation marker (mid-pregnancy).

Univariable analyses revealed that GDM (OR = 2.97, 95% CI: 1.22~7.22, *p* = 0.016), higher levels of CRP (OR = 1.30, 95% CI: 1.01~1.67, *p* = 0.042), SAA (OR = 1.33, 95% CI: 1.03~1.72, *p* = 0.032), and O_3_ exposure (OR = 1.23, 95% CI: 1.06~1.43, *p* = 0.007) were all significantly associated with increased risk of PTB. In Model 2 (Figure 1), after adjusting for maternal demographic and health-related covariates, these associations remained significant: CRP (OR = 1.31, 95% CI: 1.01~1.72, *p* = 0.049), SAA (OR = 1.37, 95% CI: 1.03~1.81, *p* = 0.028), O_3_ (OR = 1.24, 95% CI: 1.05~1.45, *p* = 0.010), and GDM (OR = 2.93, 95% CI: 1.12~7.70, *p* = 0.029). After further adjustment for environmental covariates in Model 3, CRP (OR = 1.40, 95% CI: 1.03~1.90, *p* = 0.033) and SAA (OR = 1.36, 95% CI: 1.01~1.86, *p* = 0.048) levels remained significant, while O_3_ (*p* = 0.125) and GDM (*p* = 0.090) showed marginal significance. Notably, TNF-α was identified as a significant protective factor in Model 3 (OR = 0.15, 95% CI: 0.03~0.85, *p* = 0.032) (Table S2 in the Supplement).

#### 3.2.2. Identification of Factors Influencing Low Birth Weight

**Figure 2:**
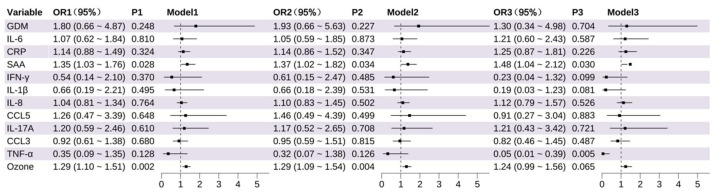
Association of Inflammatory Markers and Environmental Factors With Low Birth Weight (LBW). The odds of LBW per increase in log_2_ concentrations (and 95% CIs) of each inflammation marker (mid-pregnancy).

Using the same analytical approach, LBW was assessed as the outcome variable (Figure 2). Both univariable and multivariable results consistently indicated that higher levels of SAA were significantly associated with an increased risk of LBW (Model 3: OR = 1.48, 95% CI: 1.04~2.12, *p* = 0.030). O_3_ exposure was significantly associated with LBW in Models 1 and 2 (*p* < 0.01), but lost statistical significance in Model 3 (*p* = 0.065), possibly due to confounding by co-exposures. Notably, TNF-α emerged as a significant protective factor in Model 3 (OR = 0.05, 95% CI: 0.01~0.39, *p* = 0.005), suggesting that elevated TNF-α levels were associated with a reduced risk of LBW (Table S3 in the Supplement).

Among all biomarkers, SAA showed the most consistent association with adverse birth outcomes across models, highlighting its potential role as a key predictor.

### 3.3. Mechanistic Exploration: Metabolic and Inflammatory Pathways Linking Prenatal O_3_ Exposure to Adverse Pregnancy Outcomes

#### 3.3.1. Mediation Analysis

Based on regression results, we constructed multiple mediation models comprising both parallel and sequential mediation structures to examine whether the association between prenatal O_3_ exposure and adverse pregnancy outcomes was mediated by GDM status and maternal inflammatory biomarkers. Non-parametric bootstrapping (5000 resamples) was applied to estimate the total, direct, and indirect effects of O_3_ exposure, along with their 95% confidence intervals (CIs). GDM was specified as the first-stage mediator (M_1_), while log_2_-transformed levels of SAA, CRP, and TNF-α served as second-stage mediators (M_2_). Outcome variables included PTB and LBW.

As shown in Table 2 and Table S4, none of the indirect pathways reached statistical significance (all *p* > 0.05), and the total indirect effect accounted for less than 10% of the total effect, indicating a negligible proportion of mediation. Given the relatively small sample size (N = 235), we performed a post hoc power analysis to assess the statistical power of detecting mediating effects. The results indicated that the statistical power to detect small to medium effect sizes for the mediation pathways was lower than the conventional 0.80 threshold. This suggests that the lack of significant indirect effects could be partly due to insufficient statistical power. In contrast, the total and direct effects of O_3_ exposure on PTB and LBW remained statistically significant (*p* < 0.05), suggesting that O_3_ is more likely to exert direct effects on adverse pregnancy outcomes rather than acting through a metabolism–inflammation cascade. Although GDM and inflammatory biomarkers showed independent associations with outcomes in multivariable models, they did not form statistically significant mediating pathways between O_3_ exposure and birth outcomes. Overall, these findings suggest that the adverse effects of O_3_ on birth outcomes are predominantly direct, rather than mediated through maternal metabolic or inflammatory alterations.

#### 3.3.2. Interaction Analysis

Given that the mediation analysis did not support a causal pathway through GDM or inflammatory biomarkers in the association between prenatal O_3_ exposure and adverse pregnancy outcomes, and considering that the total and direct effects of O_3_ on PTB and LBW remained consistent across models, these findings suggest that the underlying mechanisms may not rely on a linear “metabolism–inflammation” sequential mediation pathway. Rather, they may be modified by individual differences in maternal metabolic and immune status.

To further investigate the potential moderating effects of maternal physiological states on the relationship between O_3_ exposure and birth outcomes, we constructed interaction models between O_3_ and GDM, as well as between O_3_ and individual inflammatory markers (CRP, SAA, and TNF-α), separately for PTB and LBW outcomes. These interactions were assessed using regression models incorporating interaction terms, supplemented by stratified trend plots for visual inspection of potential effect modification. O_3_ exposure was standardized to z scores prior to analysis to ensure comparability across participants. The *x*-axis in the figures represents the standardized O_3_ exposure values, which eliminates unit dependency and ensures consistency in the representation of exposure levels across plots.

**Figure 3:**
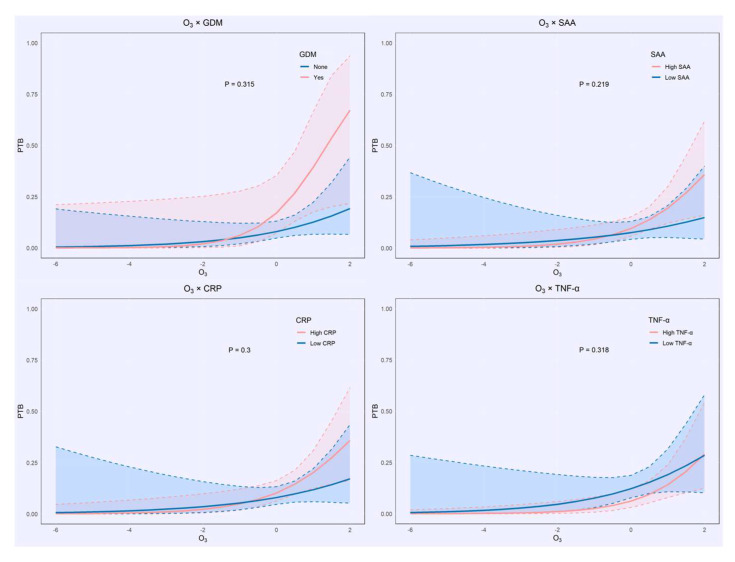
Interactive Effects of Ozone Exposure with Gestational Diabetes Mellitus (GDM) and Inflammatory Markers on the Risk of Preterm Birth. The color of the dotted lines in the figure represents different groups (blue and light blue for individuals with lower baseline inflammation levels, red and pink for individuals with higher baseline inflammation levels).

As illustrated in Figure 3 and Figure S1, none of the interaction terms reached statistical significance (all *p* > 0.05). Nevertheless, the stratified interaction plots revealed consistent, biologically plausible non-linear trends across groups.

At lower levels of O_3_ exposure, the risk of LBW was markedly higher among participants with GDM compared to those without. However, this difference diminished under higher exposure levels, suggesting that the primary effect of O_3_ may override the marginal impact of metabolic abnormalities in heavily polluted environments. Stratified analyses by CRP and SAA revealed that individuals with lower baseline inflammation levels (depicted in blue and light blue) exhibited a steeper increase in the risk of PTB and LBW in response to rising O_3_ concentrations, while those with elevated inflammation (depicted in red and pink) showed more attenuated slopes, implying a potential buffering role of chronic inflammation against exogenous oxidative stress. Similarly, the stratified curves for TNF-α aligned with its inverse association in multivariable models: among individuals with low TNF-α levels (shown in blue and light blue), higher O_3_ exposure correlated with increased risk of adverse outcomes, whereas in those with high TNF-α (shown in red and pink), the risk curves were relatively flat or even trended downward in certain exposure ranges, suggesting a protective immunomodulatory role. Although none of the interaction terms reached statistical significance, the consistent and biologically coherent trends observed across subgroups support the hypothesis that individual metabolic and immune profiles may modify the toxicological effects of prenatal O_3_ exposure.

### 3.4. Association Between Prenatal Exposure and Fetal Growth

**Figure 4:**
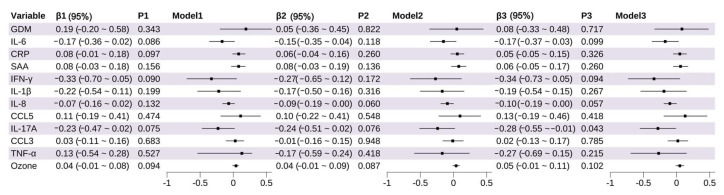
Association of Inflammatory Markers and Environmental Factors With Length-for-Age z Score (LAZ). The odds of LAZ per increase in log_2_ concentrations (and 95% CIs) of each inflammation marker (mid-pregnancy).

**Figure 5:**
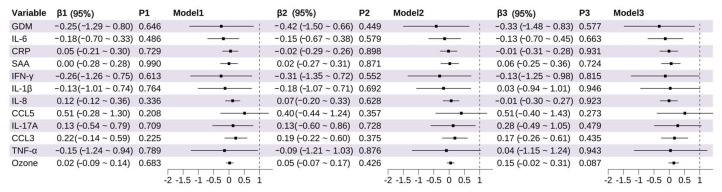
Association of Inflammatory Markers and Environmental Factors With Weight-for-Age z Score (WAZ). The odds of WAZ per increase in log_2_ concentrations (and 95% CIs) of each inflammation marker (mid-pregnancy).

Multivariable linear regression analyses were conducted using LAZ and WAZ as outcome variables. IL-17A was significantly negatively associated with LAZ (β = −0.28, 95% CI: −0.55~−0.01, *p* = 0.043) (Figure 4), while IL-8 showed a marginally significant negative correlation with LAZ (β = −0.10, 95% CI: −0.19~−0.00, *p* = 0.057). O_3_ exposure was marginally positively associated with WAZ (β = 0.15, 95% CI: −0.02~0.31, *p* = 0.087), although this association did not reach statistical significance (Figure 5).

Additionally, GDM showed no significant association with either WAZ or LAZ across all models (all *p* > 0.05). Other inflammatory markers, including IL-6, CRP, SAA, TNF-α, IFN-γ, and IL-1β, were not significantly related to LAZ or WAZ after multivariate adjustment (Supplementary Tables S5 and S6).

## 4. Discussion

This study, based on a mother–infant cohort from Ningxia, China, systematically evaluated the associations between prenatal O_3_ exposure and adverse pregnancy outcomes as well as fetal growth. It further explored potential mediation and interaction mechanisms involving maternal metabolic status and systemic inflammatory pathways. The results indicated that long-term exposure to O_3_ during pregnancy significantly increased the risks of PTB and LBW. Additionally, maternal GDM and elevated inflammatory markers were linked to adverse pregnancy outcomes. Higher maternal serum levels of SAA and CRP were associated with an increased risk of PTB, and SAA was also closely related to LBW. In contrast, TNF-α showed an inverse association with the risks of PTB and LAW. Furthermore, elevated IL-17A levels were significantly associated with reduced neonatal LAZ. These findings suggest a possible role of maternal metabolic dysfunction and inflammation in modifying the effects of prenatal O_3_ exposure on pregnancy outcomes.

We found prenatal exposure to O_3_ was significantly associated with increased risks of PTB and LBW. These associations remained robust after adjustment for maternal demographic and health characteristics, aligning with previous epidemiological findings. Multiple studies and systematic reviews have reported elevated risks of PTB associated with O_3_ exposure during pregnancy, including a 2024 Beijing cohort study which found each 10 µg/m^3^ increment in O_3_ exposure was associated with a ~3.9% higher risk of PTB [22,23,24,25,26]. The adverse effects of O_3_ on fetal weight have similarly been documented across diverse populations [27,28,29]. A meta-analysis of six studies estimated that each 10 ppb increase in O_3_ was associated with a reduction in birth weight ranging from 4.6 to 27.3 g [30]. A study from Guangzhou further identified gestational weeks 15 to 26 as a critical window for O_3_ exposure in relation to LBW [31]. Recent cohort evidence from China demonstrated that combined prenatal exposure to O_3_ and PM_2.5_ was associated with a significantly higher risk of LBW compared with exposure to either pollutant alone, suggesting a synergistic effect of the pollutant mixture [32]. Additionally, in this study, the observed associations between O_3_ and adverse outcomes were attenuated after controlling for co-exposure to other pollutants such as PM_2.5_ and NO_2_, further suggesting that the effects of O_3_ may not be entirely independent. A birth cohort study in Finland found that simultaneous exposure to high levels of PM_2.5_ and O_3_ during pregnancy increased the incidence of PTB to 24.2%, with an excess risk attributable to their interaction reaching 230%, substantially greater than the additive effects of each pollutant alone, indicating a potential synergistic mechanism between them [33]. These findings may partly explain the attenuation of O_3_ effects in our multi-pollutant models and highlight the limitations of conventional single-pollutant approaches in capturing the complexity of real-world environmental exposures. They also underscore the importance of incorporating multi-pollutant models in future research on prenatal environmental health.

As a key manifestation of maternal metabolic dysregulation, GDM was significantly associated with increased PTB risk in this study. In univariate analysis, GDM markedly elevated the risk of PTB, and this association remained marginally significant in multivariable models. These findings support the role of GDM as an independent risk factor for PTB, potentially mediated by mechanisms involving induction of iNOS expression, which accelerates cervical maturation leading to preterm labor [34]. Although GDM did not demonstrate a significant mediating effect on the pathway between O_3_ exposure and PTB in this study, it may amplify the toxic effects of O_3_ by exacerbating oxidative stress and inflammatory responses. In addition, no significant association was observed between GDM and LBW in this study. However, previous prospective cohort studies have reported that GDM was strongly associated with increased risks of macrosomia and large-for-gestational-age (LGA) fetuses [35], suggesting that its impact on fetal growth may primarily manifest as increased birth weight.

Existing research suggests that O_3_ can induce oxidative stress and insulin resistance, contributing to systemic metabolic disturbances, impaired placental perfusion, and intrauterine inflammation. These mechanisms may collectively increase the risk of adverse pregnancy outcomes. Findings from both human cohort studies and animal experiments support this mechanism, suggesting that O_3_ exposure may increase the risk of PTB and LBW through these pathways [36,37]. In the analysis of inflammatory biomarkers, elevated maternal serum levels of SAA were significantly associated with increased risks of both PTB and LBW. These associations remained robust after adjustment for multiple layers of covariates, supporting the role of SAA as an independent risk factor for adverse pregnancy outcomes. Recent studies have reinforced this notion, with a 2024 observational study from Romania highlighting that elevated maternal serum SAA levels (≥15 mg/L) were strongly associated with a nearly 29-fold increase in the risk of PTB, even after controlling for confounding factors such as maternal age, history of PTB, and other pregnancy-related conditions [38,39]. CRP levels were also positively associated with PTB but showed no significant association with LBW, which is inconsistent with some previous findings. Prior evidence suggests that CRP levels exceeding 4 mg/L may serve as a predictor of idiopathic PTB, and levels above 2 mg/L may be positively associated with LBW [40]. The absence of a significant association between CRP and LBW in the present study may reflect population heterogeneity or limited statistical power. Interestingly, TNF-α levels were inversely associated with the risks of PTB and LBW in this cohort, indicating a potential protective effect in multivariable models. However, the possibility of small-sample or context-specific effects should be considered when interpreting this inverse association. This finding diverges from existing literature, which generally links elevated TNF-α concentrations to a higher risk of adverse pregnancy outcomes, including hypertensive disorders, recurrent miscarriage, and fetal growth restriction [41,42]. One possible explanation for this divergent finding could be the presence of compensatory anti-inflammatory mechanisms. In response to the oxidative stress induced by prenatal ozone exposure, elevated TNF-α might trigger a protective immune response, potentially mitigating the harmful effects of inflammation and reducing the risks of PTB and LBW. Moreover, the physiological heterogeneity in immune responses across individuals may further explain the observed inverse relationship [43]. This suggests that immune modulation, rather than a direct inflammatory effect, could play a key role in shaping the outcomes, particularly in cases of immune compensation to environmental stressors. To validate these hypotheses, further research exploring these mechanisms in larger and more diverse cohorts is needed to confirm these findings and better understand the role of TNF-α in pregnancy. Moreover, although SAA and CRP were each significantly associated with adverse pregnancy outcomes in primary analyses, mediation models did not identify statistically significant indirect effects linking these factors to O_3_ exposure. This finding is inconsistent with other studies, which have reported enhanced inflammatory responses that may disrupt placental function and harm fetal growth and development [44,45,46]. We need a larger sample size to confirm its results. Notably, SAA, a sensitive acute-phase reactant, is readily activated following O_3_ exposure, triggering systemic inflammation that damages placental vascular structure, impairs perfusion, and increases the risk of premature rupture of membranes and uterine contractions, and has been suggested as a potential biomarker of inflammation and oxidative stress during pregnancy [47]. Moreover, previous studies have identified maternal IL-1β and TNF-α as mediators in the association between prenatal exposure to environmental tobacco smoke and reduced birth weight [48]. However, similar pathways were not observed in this study, possibly due to limited sample size, weak associations, or collinearity between inflammatory biomarkers, which may have affected the stability of mediation estimates.

Regarding fetal growth, this study identified a significant negative association between maternal IL-17A levels and LAZ. IL-17A, a cytokine produced by T-helper 17 (Th17) cells and other immune cells, plays a critical role in defending against various microbial pathogens. Emerging evidence has highlighted the regulatory role of IL-17A in placental function and intrauterine homeostasis, particularly through its influence on the expression of glucose transporters. By modulating the expression of these transporters, IL-17A may impair placental glucose uptake, reducing placental efficiency and limiting fetal nutrient supply, which could contribute to decreased fetal growth and development [49]. Previous studies have reported associations between IL-17A levels and both PTB and LBW [50]; however, its relationship with fetal growth parameters has not been previously described, highlighting the novelty and exploratory value of the present findings in this field.

This study has several strengths. It represents the first mother–infant cohort established in Northwest China to systematically evaluate the effects of prenatal O_3_ exposure on PTB, LBW, and fetal growth. It also examined the potential mediating and interactive roles of maternal GDM and inflammatory biomarkers in these associations. By integrating air pollution, inflammation, and metabolism, the study developed a novel multi-pathway and multi-interaction analytical framework, enabling a more comprehensive assessment of the interplay between environmental exposures and maternal immune–metabolic status, and providing a theoretical basis for personalized pregnancy risk management.

There are some limitations to this study. Firstly, the study population was restricted to a single geographic region, which may limit the generalizability of the findings. The relatively small sample size may have reduced the statistical power of the mediation and interaction analyses. Secondly, inflammatory biomarkers were measured only once during mid-pregnancy without longitudinal follow-up, limiting the ability to capture dynamic immune changes across gestation. Thirdly, the outcomes assessed were confined to short-term birth indicators, precluding evaluation of the long-term effects of prenatal O_3_ exposure on postnatal growth, metabolic health, and neurodevelopment. Moreover, co-exposure to other pollutants was not examined, which may hinder a comprehensive understanding of real-world environmental exposure scenarios. Additionally, residual confounding by unmeasured socioeconomic factors cannot be excluded.

## 5. Conclusions

This study results indicate that prenatal O_3_ exposure independently increases the risk of PTB and LBW, while maternal metabolic and inflammatory alterations contribute to but do not mediate these effects. The findings underscore O_3_ as a critical environmental risk factor during pregnancy and highlight the need to account for individual immunometabolic susceptibility in prenatal risk assessment. Future research should involve larger, multi-center mother–infant cohorts, incorporate longitudinal biomarker monitoring to capture immune and metabolic dynamics across gestation, and evaluate long-term offspring outcomes. Moreover, to gain a deeper understanding of the timing and effects of prenatal O_3_ exposure, future studies should prioritize trimester-specific exposure analyses. Integrating multi-pollutant exposure models with biomarker profiling will be essential for identifying potential synergistic effects and refining prenatal environmental risk assessment frameworks to inform targeted public health interventions.

## Figures and Tables

**Table 1:** Characteristics of the Study Population During Their Second Trimester by Preterm Birth Status.

Characteristic	Frequency (N) (Proportion, %)			
	Overall (N = 235)	Term Births [n = 208 (88.51%)]	Preterm Births [n = 27 (11.49%)]	*p*
Age at Delivery, M (Q_1_, Q_3_)	31.00 (29.00, 34.00)	31.00 (29.00, 33.00)	30.00 (28.00, 35.00)	0.836
Gestational Weight Gain, M (Q_1_, Q_3_)	5.50 (3.00, 8.00)	5.05 (3.00, 8.00)	6.00 (3.00, 10.00)	0.648
Pre-pregnancy BMI, M (Q_1_, Q_3_)	21.51 (19.72, 23.62)	21.44 (19.55, 23.48)	22.27 (21.12, 24.06)	0.199
O_3_, M (Q_1_, Q_3_)	108.70 (106.47, 109.87)	108.60 (106.37, 109.81)	109.17 (107.37, 110.60)	0.053
Pre-pregnancy Health				0.453
Good	151 (64.26)	133 (63.94)	18 (66.67)	
General	80 (34.04)	72 (34.62)	8 (29.63)	
Bad	4 (1.70)	3 (1.44)	1 (3.70)	
Primiparity				0.833
None	135 (57.45)	120 (57.69)	15 (55.56)	
Yes	100 (42.55)	88 (42.31)	12 (44.44)	
Mode of Conception				0.666
Natural Conception	218 (92.77)	194 (93.27)	24 (88.89)	
Assisted Reproduction	17 (7.23)	14 (6.73)	3 (11.11)	
Season of Conception				0.900
Spring	34 (14.47)	31 (14.90)	3 (11.11)	
Summer	1 (0.43)	1 (0.48)	0 (0.00)	
Autumn	98 (41.70)	87 (41.83)	11 (40.74)	
Winter	102 (43.40)	89 (42.79)	13 (48.15)	
Education Level				0.878
Junior High School or Below	18 (7.66)	16 (7.69)	2 (7.41)	
High School	38 (16.17)	35 (16.83)	3 (11.11)	
Bachelor’s Degree	157 (66.81)	138 (66.35)	19 (70.37)	
Master’s Degree or Above	22 (9.36)	19 (9.13)	3 (11.11)	
Careers				0.303
Enterprises and public institutions	91 (38.72)	83 (39.90)	8 (29.63)	
Other careers	144 (61.28)	125 (60.10)	19 (70.37)	
Per Capita Monthly Income (RMB)				0.867
Low	14 (5.96)	12 (5.77)	2 (7.41)	
Medium	124 (52.77)	109 (52.40)	15 (55.56)	
High	97 (41.28)	87 (41.83)	10 (37.04)	
Address				0.805
City	210 (89.36)	185 (88.94)	25 (92.59)	
Rural	25 (10.64)	23 (11.06)	2 (7.41)	
Sleep Quality				0.328
Very Good	48 (20.43)	43 (20.67)	5 (18.52)	
Fairly Good	140 (59.57)	126 (60.58)	14 (51.85)	
Fairly Poor	44 (18.72)	37 (17.79)	7 (25.93)	
Very Poor	3 (1.28)	2 (0.96)	1 (3.70)	
Sleep Efficiency				0.110
>85%	174 (74.04)	156 (75.00)	18 (66.67)	
75~84%	43 (18.30)	39 (18.75)	4 (14.81)	
65~74%	15 (6.38)	11 (5.29)	4 (14.81)	
<65%	3 (1.28)	2 (0.96)	1 (3.70)	
Sleep Disturbance				0.770
None	20 (8.51)	19 (9.13)	1 (3.70)	
Low	171 (72.77)	150 (72.12)	21 (77.78)	
Medium	43 (18.30)	38 (18.27)	5 (18.52)	
High	1 (0.43)	1 (0.48)	0 (0.00)	

Note: *p* < 0.05 means a significant difference. Q1: 1st Quartile, Q3: 3st Quartile.

**Table 2:** Mediating Role of Gestational Diabetes Mellitus (GDM) and Inflammatory Markers in the Association Between Ozone Exposure and Preterm Birth.

	Path Structure	Effect	Boot SE	BootLLCI	BootULCI	*p*
Indirect Effect	O_3_⇒GDM⇒PTB	0.000	0.014	−0.024	0.035	0.985
	O_3_⇒Log_2_SAA⇒PTB	0.001	0.013	−0.006	0.043	0.929
	O_3_⇒GDM⇒Log_2_SAA⇒PTB	0.000	0.001	−0.003	0.003	0.990
Direct Effect	O_3_⇒PTB	0.015	0.006	0.002	0.028	0.021
Total Effect	O_3_⇒PTB	0.016	0.007	0.004	0.029	0.012
Indirect Effect	O_3_⇒GDM⇒PTB	0.000	0.013	−0.024	0.033	0.985
	O_3_⇒Log_2_CRP⇒PTB	0.000	0.008	−0.008	0.024	0.969
	O_3_⇒GDM⇒Log_2_CRP⇒PTB	0.000	0.002	−0.003	0.005	0.989
Direct Effect	O_3_⇒PTB	0.016	0.006	0.003	0.028	0.014
Total Effect	O_3_⇒PTB	0.016	0.007	0.004	0.029	0.012
Indirect Effect	O_3_⇒GDM⇒PTB	0.000	0.014	−0.025	0.034	0.985
	O_3_⇒Log_2_TNF-α⇒PTB	−0.001	0.008	−0.024	0.006	0.933
	O_3_⇒GDM⇒Log_2_TNF-α⇒PTB	0.000	0.001	−0.002	0.002	0.989
Direct Effect	O_3_⇒PTB	0.017	0.006	0.004	0.029	0.010
Total Effect	O_3_⇒PTB	0.016	0.007	0.004	0.029	0.012

Note: BootLLCI refers to the lower limit of the 95% confidence interval obtained via bootstrap sampling, and BootULCI refers to the upper limit. Boot SE represents the standard error of the indirect effect, estimated from 5000 bootstrap resamples.

## Data Availability

Data will be made available on request.

## Supplementary Materials

The following supporting information can be downloaded at: https://www.mdpi.com/article/10.3390/toxics13110983/s1, Figure S1: Interactive Effects of Ozone Exposure with Gestational Diabetes Mellitus (GDM) and Inflammatory Markers on the Risk of Low Birth Weight; Table S1: Characteristics of the Study Population During Their Second Trimester by Low Birth Weight Status; Table S2: Association of Inflammatory Markers and Environmental Factors With Preterm Birth (PTB). Table S3: Association of Inflammatory Markers and Environmental Factors With Low Birth Weight (LBW); Table S4: Mediating Role of Gestational Diabetes Mellitus (GDM) and Inflammatory Markers in the Association Between Ozone Exposure and Low Birth Weight. Table S5: Association of Inflammatory Markers and Environmental Factors With Length-for-Age z Score (LAZ); Table S6: Association of Inflammatory Markers and Environmental Factors With Wength-for-Age z Score (WAZ).
