# Supplementary material for: Association of Prenatal Ozone Exposure with Fetal Growth and Birth Outcomes: Roles of Maternal Inflammation and Metabolic Dysregulation
Source: Toxics. 2025 Nov 15;13(11):983. doi: 10.3390/toxics13110983 (PMC12656322; doi:10.3390/toxics13110983)
Supplement: Supplementary file 1 [file toxics-13-00983-s001.zip › toxics-3961458-Supplementary Materials.pdf]

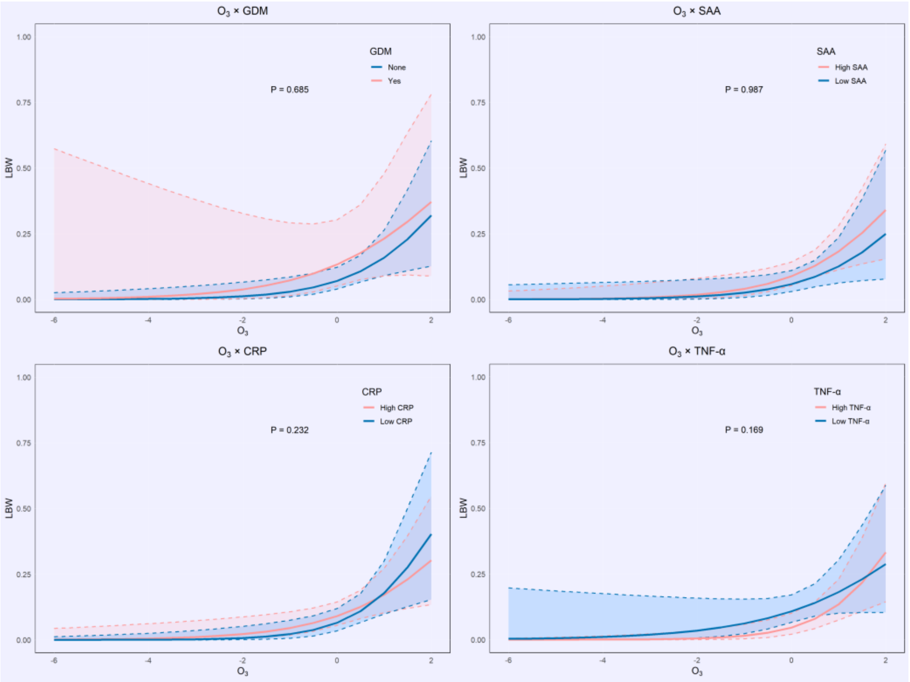

Figure S1. Interactive Effects of Ozone Exposure with Gestational Diabetes Mellitus (GDM) and Inflammatory Markers on the Risk of Low Birth Weight. The color of the dotted lines in the figure represents different groups (blue and light blue for individuals with lower baseline inflammation levels, red and pink for individuals with higher baseline inflammation levels).

Table S1. Characteristics of the Study Population During Their Second Trimester by Low Birth Weight Status

Characteristic	Frequency (N) (proportion, %)			*P*
	Overall (N = 235)	Normal Weight [n = 211(89.79%)]	Low Birth Weight [n = 24(10.21%)]	
Age at Delivery, M (Q_1, Q_3)	31.00 (29.00, 34.00)	31.00 (29.00, 33.00)	30.50 (28.00, 35.00)	0.810
Gestational Weight Gain, M (Q_1, Q_3)	5.50 (3.00, 8.00)	5.50 (3.00, 8.00)	5.75 (3.00, 8.88)	0.844
Pre-pregnancy BMI, M (Q_1, Q_3)	21.51 (19.72, 23.62)	21.48 (19.64, 23.61)	22.16 (20.65, 24.02)	0.544
O_3, M (Q_1, Q_3)	108.70 (106.47, 109.87)	108.57 (106.37, 109.77)	109.63 (108.66, 111.63)	0.005
Pre-pregnancy Health				0.360
Good	151 (64.26)	137 (64.93)	14 (58.33)	
General	80 (34.04)	71 (33.65)	9 (37.50)	
Bad	4 (1.70)	3 (1.42)	1 (4.17)	
Primiparity				0.732
None	135 (57.45)	122 (57.82)	13 (54.17)	
Yes	100 (42.55)	89 (42.18)	11 (45.83)	
Mode of Conception				1.000
Natural Conception	218 (92.77)	196 (92.89)	22 (91.67)	
Assisted Reproduction	17 (7.23)	15 (7.11)	2 (8.33)	
Season of Conception				0.821
Spring	34 (14.47)	30 (14.22)	4 (16.67)	
Summer	1 (0.43)	1 (0.47)	0 (0.00)	
Autumn	98 (41.70)	87 (41.23)	11 (45.83)	
Winter	102 (43.40)	93 (44.08)	9 (37.50)	
Education Level				0.831
Junior High School or Below	18 (7.66)	17 (8.06)	1 (4.17)	
High School	38 (16.17)	35 (16.59)	3 (12.50)	
Bachelor's Degree	157 (66.81)	140 (66.35)	17 (70.83)	
Master's Degree or Above	22 (9.36)	19 (9.00)	3 (12.50)	

Characteristic	Frequency (N) (proportion, %)			P
	Overall (N = 235)	Normal Weight [n = 211(89.79%)]	Low Birth Weight [n = 24(10.21%)]	
Careers				0.567
Enterprises and public institutions	91 (38.72)	83 (39.34)	8 (33.33)	
Other careers	144 (61.28)	128 (60.66)	16 (66.67)	
Per Capita Monthly Income (RMB)				0.926
Low	14 (5.96)	13 (6.16)	1 (4.17)	
Medium	124 (52.77)	111 (52.61)	13 (54.17)	
High	97 (41.28)	87 (41.23)	10 (41.67)	
Address				0.970
City	210 (89.36)	188 (89.10)	22 (91.67)	
Rural	25 (10.64)	23 (10.90)	2 (8.33)	
Sleep Quality				0.474
Very Good	48 (20.43)	43 (20.38)	5 (20.83)	
Fairly Good	140 (59.57)	127 (60.19)	13 (54.17)	
Fairly Poor	44 (18.72)	39 (18.48)	5 (20.83)	
Very Poor	3 (1.28)	2 (0.95)	1 (4.17)	
Sleep Efficiency				0.394
>85%	174 (74.04)	157 (74.41)	17 (70.83)	
75~84%	43 (18.30)	39 (18.48)	4 (16.67)	
65~74%	15 (6.38)	13 (6.16)	2 (8.33)	
<65%	3 (1.28)	2 (0.95)	1 (4.17)	
Sleep Disturbance				0.789
None	20 (8.51)	18 (8.53)	2 (8.33)	
Low	171 (72.77)	152 (72.04)	19 (79.17)	
Medium	43 (18.30)	40 (18.96)	3 (12.50)	
High	1 (0.43)	1 (0.47)	0 (0.00)	

Note: $p < 0.05$ means a significant difference. Q1: 1st Quartile, Q3: 3st Quartile.

Table S2. Association of Inflammatory Markers and Environmental Factors With Preterm Birth (PTB)

Variables	Model1		Model2		Model3	
	**OR (95%CI)**	***P***	**OR (95%CI)**	***P***	**OR (95%CI)**	***P***
GDM						
None	1.00 (Reference)		1.00 (Reference)		1.00 (Reference)	
Yes	2.97 (1.22 ~ 7.22)	0.016	2.93 (1.12 ~ 7.70)	0.029	2.61 (0.86 ~ 7.92)	0.090
Log_2IL-6	1.05 (0.63 ~ 1.77)	0.843	1.08 (0.62 ~ 1.87)	0.797	1.09 (0.58 ~ 2.03)	0.792
Log_2CRP	1.30 (1.01 ~ 1.67)	0.042	1.31 (1.01 ~ 1.72)	0.049	1.40 (1.03 ~ 1.90)	0.033
Log_2SAA	1.33 (1.03 ~ 1.72)	0.032	1.37 (1.03 ~ 1.81)	0.028	1.36 (1.01 ~ 1.86)	0.048
Log_2IFN-γ	0.44 (0.12 ~ 1.71)	0.239	0.40 (0.09 ~ 1.76)	0.227	0.22 (0.04 ~ 1.22)	0.084
Log_2IL-1β	0.67 (0.22 ~ 2.09)	0.491	0.62 (0.18 ~ 2.20)	0.462	0.41 (0.09 ~ 1.83)	0.243
Log_2IL-8	0.93 (0.72 ~ 1.21)	0.594	0.89 (0.67 ~ 1.20)	0.459	0.89 (0.65 ~ 1.23)	0.494
Log_2CCL5	0.74 (0.37 ~ 1.48)	0.391	0.74 (0.36 ~ 1.53)	0.413	0.57 (0.24 ~ 1.37)	0.210
Log_2IL-17A	0.82 (0.41 ~ 1.63)	0.571	0.73 (0.32 ~ 1.66)	0.450	0.76 (0.31 ~ 1.88)	0.554
Log_2CCL3	0.92 (0.63 ~ 1.35)	0.662	0.82 (0.52 ~ 1.29)	0.400	0.80 (0.49 ~ 1.32)	0.384
Log_2TNF-α	0.40 (0.11 ~ 1.44)	0.161	0.34 (0.08 ~ 1.42)	0.139	0.15 (0.03 ~ 0.85)	0.032
O_3	1.23 (1.06 ~ 1.43)	0.007	1.24 (1.05 ~ 1.45)	0.010	1.18 (0.95 ~ 1.47)	0.125

Note: OR: Odds Ratio, CI: Confidence Interval

Model1: Crude

Model2: Adjust: Age at Delivery, Season of Conception, Primiparity, Pre-pregnancy Health, Sleep Quality,Pre-pregnancy BMI, Gestational Weight Gain

Model3: Adjust: Age at Delivery, Season of Conception, Primiparity, Pre-pregnancy Health, Sleep Quality,Pre-pregnancy BMI, Gestational Weight Gain, Address, Education Level, Per Capita Monthly Income, APGAR, ALAN, NDVI, NO_2, $PM_{2.5}$, Rainfall, Temperature

Table S3. Association of Inflammatory Markers and Environmental Factors With Low Birth Weight (LBW)

Variables	Model1		Model2		Model3	
	OR (95%CI)	*P*	OR (95%CI)	*P*	OR (95%CI)	*P*
GDM						
None	1.00 (Reference)		1.00 (Reference)		1.00 (Reference)	
Yes	1.80 (0.66 ~ 4.87)	0.248	1.93 (0.66 ~ 5.63)	0.227	1.30 (0.34 ~ 4.98)	0.704
Log_2IL-6	1.07 (0.62 ~ 1.84)	0.810	1.05 (0.59 ~ 1.85)	0.873	1.21 (0.60 ~ 2.43)	0.587
Log_2CRP	1.14 (0.88 ~ 1.49)	0.324	1.14 (0.86 ~ 1.52)	0.347	1.25 (0.87 ~ 1.81)	0.226
Log_2SAA	1.35 (1.03 ~ 1.76)	0.028	1.37 (1.02 ~ 1.82)	0.034	1.48 (1.04 ~ 2.12)	0.030
Log_2IFN-γ	0.54 (0.14 ~ 2.10)	0.370	0.61 (0.15 ~ 2.47)	0.485	0.23 (0.04 ~ 1.32)	0.099
Log_2IL-1β	0.66 (0.19 ~ 2.21)	0.495	0.66 (0.18 ~ 2.39)	0.531	0.19 (0.03 ~ 1.23)	0.081
Log_2IL-8	1.04 (0.81 ~ 1.34)	0.764	1.10 (0.83 ~ 1.45)	0.502	1.12 (0.79 ~ 1.57)	0.526
Log_2CCL5	1.26 (0.47 ~ 3.39)	0.648	1.46 (0.49 ~ 4.39)	0.499	0.91 (0.27 ~ 3.04)	0.883
Log_2IL-17A	1.20 (0.59 ~ 2.46)	0.610	1.17 (0.52 ~ 2.65)	0.708	1.21 (0.43 ~ 3.42)	0.721
Log_2CCL3	0.92 (0.61 ~ 1.38)	0.680	0.95 (0.59 ~ 1.51)	0.815	0.82 (0.46 ~ 1.45)	0.487
Log_2TNF-α	0.35 (0.09 ~ 1.35)	0.128	0.32 (0.07 ~ 1.38)	0.126	0.05 (0.01 ~ 0.39)	0.005
O_3	1.29 (1.10 ~ 1.51)	0.002	1.29 (1.09 ~ 1.54)	0.004	1.24 (0.99 ~ 1.56)	0.065

Note: OR: Odds Ratio, CI: Confidence Interval

Model1: Crude

Model2: Adjust: Age at Delivery, Season of Conception, Primiparity, Pre-pregnancy Health, Sleep Quality,Pre-pregnancy BMI, Gestational Weight Gain

Model3: Adjust: Age at Delivery, Season of Conception, Primiparity, Pre-pregnancy Health, Sleep Quality,Pre-pregnancy BMI, Gestational Weight Gain, Address, Education Level, Per Capita Monthly Income, APGAR, ALAN, NDVI, NO_2, $PM_{2.5}$, Rainfall, Temperature

Table S4. Mediating Role of Gestational Diabetes Mellitus (GDM) and Inflammatory Markers in the Association Between Ozone Exposure and Low Birth Weight

	**Path Structure**	**Effect**	**Boot SE**	**BootLLCI**	**BootULCI**	***P***
Indirect Effect	O_3⇒GDM⇒LBW	0.000	0.009	-0.017	0.020	0.990
	O_3⇒Log_2SAA⇒LBW	0.001	0.013	-0.005	0.045	0.928
	O_3⇒GDM⇒Log_2SAA⇒LBW	0.000	0.001	-0.003	0.003	0.990
Direct Effect	O_3⇒LBW	0.017	0.006	0.005	0.029	0.008
Total Effect	O_3⇒LBW	0.018	0.006	0.006	0.030	0.004
Indirect Effect	O_3⇒GDM⇒LBW	0.000	0.009	-0.017	0.020	0.989
	O_3⇒Log_2CRP⇒LBW	0.000	0.005	-0.008	0.013	0.979
	O_3⇒GDM⇒Log_2CRP⇒LBW	0.000	0.001	-0.002	0.002	0.992
Direct Effect	O_3⇒LBW	0.018	0.006	0.006	0.030	0.005
Total Effect	O_3⇒LBW	0.018	0.006	0.006	0.030	0.004
Indirect Effect	O_3⇒GDM⇒LBW	0.000	0.008	-0.017	0.020	0.989
	O_3⇒Log_2TNF-α⇒LBW	-0.001	0.009	-0.027	0.008	0.933
	O_3⇒GDM⇒Log_2TNF-α⇒LBW	0.000	0.001	-0.002	0.003	0.989
Direct Effect	O_3⇒LBW	0.019	0.006	0.006	0.031	0.003
Total Effect	O_3⇒LBW	0.018	0.006	0.006	0.030	0.004

Note：BootLLCI refers to the lower limit of the 95% confidence interval obtained via bootstrap sampling, and BootULCI refers to the upper limit. Boot SE represents the standard error of the indirect effect, estimated from 5,000 bootstrap resamples.

Table S5. Association of Inflammatory Markers and Environmental Factors With Length-for-Age z Score (LAZ)

Variables	Model1		Model2		Model3	
	**β (95%CI)**	***P***	**β(95%CI)**	***P***	**β (95%CI)**	***P***
GDM						
0	1.00 (Reference)		1.00 (Reference)		1.00 (Reference)	
1	0.19 (-0.20 ~ 0.58)	0.343	0.05 (-0.36 ~ 0.45)	0.822	0.08 (-0.33 ~ 0.48)	0.717
Log_2IL-6	-0.17 (-0.36 ~ 0.02)	0.086	-0.15 (-0.35 ~ 0.04)	0.118	-0.17 (-0.37 ~ 0.03)	0.099
Log_2CRP	0.08 (-0.01 ~ 0.18)	0.097	0.06 (-0.04 ~ 0.16)	0.260	0.05 (-0.05 ~ 0.15)	0.326
Log_2SAA	0.08 (-0.03 ~ 0.18)	0.156	0.08 (-0.03 ~ 0.19)	0.136	0.06 (-0.05 ~ 0.17)	0.260
Log_2IFN-γ	-0.33 (-0.70 ~ 0.05)	0.090	-0.27 (-0.65 ~ 0.12)	0.172	-0.34 (-0.73 ~ 0.05)	0.094
Log_2IL-1β	-0.22 (-0.54 ~ 0.11)	0.199	-0.17 (-0.50 ~ 0.16)	0.316	-0.19 (-0.54 ~ 0.15)	0.267
Log_2IL-8	-0.07 (-0.16 ~ 0.02)	0.132	-0.09 (-0.19 ~ 0.00)	0.060	-0.10 (-0.19 ~ 0.00)	0.057
Log_2CCL5	0.11 (-0.19 ~ 0.41)	0.474	0.10 (-0.22 ~ 0.41)	0.548	0.13 (-0.19 ~ 0.46)	0.418
Log_2IL-17A	-0.23 (-0.47 ~ 0.02)	0.075	-0.24 (-0.51 ~ 0.02)	0.076	-0.28 (-0.55 ~ -0.01)	0.043
Log_2CCL3	0.03 (-0.11 ~ 0.16)	0.683	-0.01 (-0.16 ~ 0.15)	0.948	0.02 (-0.13 ~ 0.17)	0.785
Log_2TNF-α	0.13 (-0.54 ~ 0.28)	0.527	-0.17 (-0.59 ~ 0.24)	0.418	-0.27 (-0.69 ~ 0.15)	0.215
O_3	0.04 (-0.01 ~ 0.08)	0.094	0.04 (-0.01 ~ 0.09)	0.087	0.05 (-0.01 ~ 0.11)	0.102

Note: CI: Confidence Interval

Model1: Crude

Model2: Adjust: Age at Delivery, Season of Conception, Primiparity, Pre-pregnancy Health, Sleep Quality,Pre-pregnancy BMI, Gestational Weight Gain

Model3: Adjust: Age at Delivery, Season of Conception, Primiparity, Pre-pregnancy Health, Sleep Quality,Pre-pregnancy BMI, Gestational Weight Gain, Address, Education Level, Per Capita Monthly Income, APGAR, ALAN, NDVI, NO_2, $PM_{2.5}$, Rainfall, Temperature

Table S6. Association of Inflammatory Markers and Environmental Factors With Wength-for-Age z Score (WAZ)

Variables	Model1		Model2		Model3	
	**β (95%CI)**	***P***	**β (95%CI)**	***P***	**β (95%CI)**	***P***
GDM						
0	1.00 (Reference)		1.00 (Reference)		1.00 (Reference)	
1	-0.25 (-1.29 ~ 0.80)	0.646	-0.42 (-1.50 ~ 0.66)	0.449	-0.33 (-1.48 ~ 0.83)	0.577
Log_2IL-6	-0.18 (-0.70 ~ 0.33)	0.486	-0.15 (-0.67 ~ 0.38)	0.579	-0.13 (-0.70 ~ 0.45)	0.663
Log_2CRP	0.05 (-0.21 ~ 0.30)	0.729	-0.02 (-0.29 ~ 0.26)	0.898	-0.01 (-0.31 ~ 0.28)	0.931
Log_2SAA	0.00 (-0.28 ~ 0.28)	0.990	0.02 (-0.27 ~ 0.31)	0.871	0.06 (-0.25 ~ 0.36)	0.724
Log_2IFN-γ	-0.26 (-1.26 ~ 0.75)	0.613	-0.31 (-1.35 ~ 0.72)	0.552	-0.13 (-1.25 ~ 0.98)	0.815
Log_2IL-1β	-0.13 (-1.01 ~ 0.74)	0.764	-0.18 (-1.07 ~ 0.71)	0.692	0.03 (-0.94 ~ 1.01)	0.946
Log_2IL-8	0.12 (-0.12 ~ 0.36)	0.336	0.07 (-0.20 ~ 0.33)	0.628	-0.01 (-0.30 ~ 0.27)	0.923
Log_2CCL5	0.51 (-0.28 ~ 1.30)	0.208	0.40 (-0.44 ~ 1.24)	0.357	0.51 (-0.40 ~ 1.43)	0.273
Log_2IL-17A	0.13 (-0.54 ~ 0.79)	0.709	0.13 (-0.60 ~ 0.86)	0.728	0.28 (-0.49 ~ 1.05)	0.479
Log_2CCL3	0.22 (-0.14 ~ 0.59)	0.225	0.19 (-0.22 ~ 0.60)	0.375	0.17 (-0.26 ~ 0.61)	0.435
Log_2TNF-α	-0.15 (-1.24 ~ 0.94)	0.789	-0.09 (-1.21 ~ 1.03)	0.876	0.04 (-1.15 ~ 1.24)	0.943
O_3	0.02 (-0.09 ~ 0.14)	0.683	0.05 (-0.07 ~ 0.17)	0.426	0.15 (-0.02 ~ 0.31)	0.087

Note: CI: Confidence Interval

Model1: Crude

Model2: Adjust: Age at Delivery, Season of Conception, Primiparity, Pre-pregnancy Health, Sleep Quality,Pre-pregnancy BMI, Gestational Weight Gain

Model3: Adjust: Age at Delivery, Season of Conception, Primiparity, Pre-pregnancy Health, Sleep Quality,Pre-pregnancy BMI, Gestational Weight Gain, Address, Education Level, Per Capita Monthly Income, APGAR, ALAN, NDVI, NO_2, $PM_{2.5}$, Rainfall, Temperature
